# Supplementary material for: A systematic review of worldwide causal and correlational evidence on digital media and democracy
Source: Nat Hum Behav. 2022 Nov 7;7(1):74–101. doi: 10.1038/s41562-022-01460-1 (PMC9883171; doi:10.1038/s41562-022-01460-1)
Supplement: Supplementary file 1 — Supplementary Figs. 1–6, Tables 1 and 2, Info box and references. [file 41562_2022_1460_MOESM1_ESM.pdf]

nature human behaviour

Article

https://doi.org/10.1038/s41562-022-01460-1

A systematic review of worldwide causal and correlational evidence on digital media and democracy

In the format provided by the authors and unedited

Supplementary material for:
Digital Media and Democracy: A Systematic Review of Causal and Correlational Evidence Worldwide

Philipp Lorenz-Spreen*,† Lisa Oswald* Stephan Lewandowsky
Ralph Hertwig
*these authors contributed equally; †lorenz-spreen@mpib-berlin.mpg.de

July 12, 2022

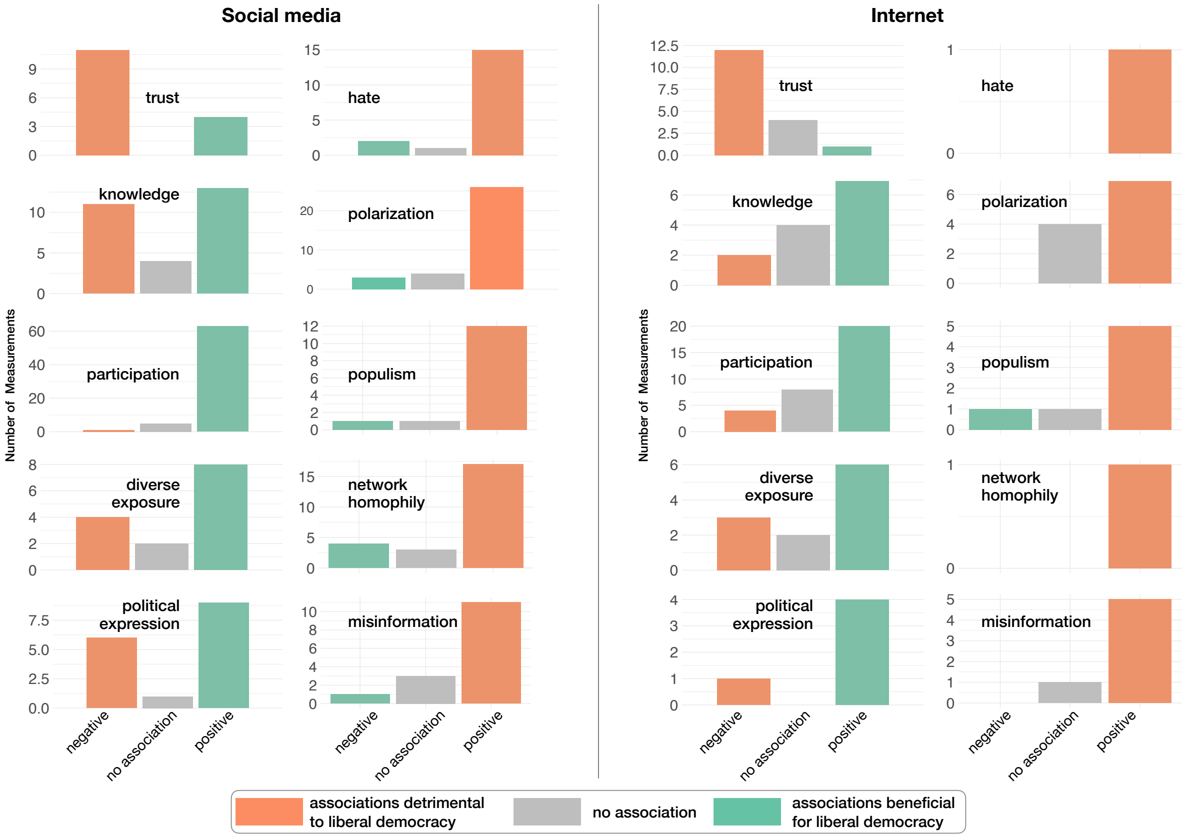

Supplementary Figure 1: Distribution of directions of associations from the full sample, reported for various political variables (see Fig. 7d for a breakdown). Split between digital media variables that describe social media vs. internet use more generally.

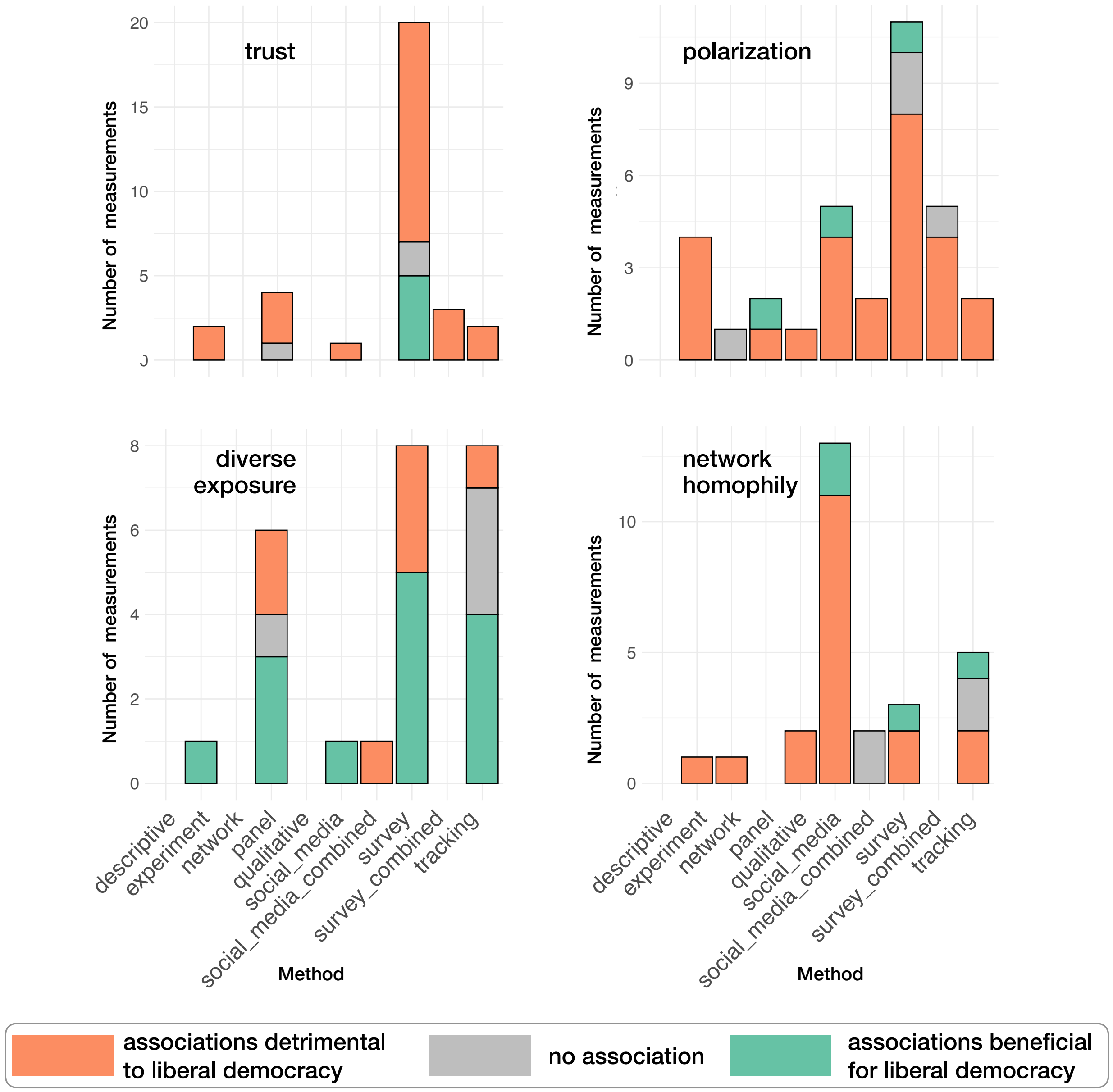

Supplementary Figure 2: Distribution of directions of associations from the full sample, reported for various political variables. Insets show exemplary the distribution of associations with trust, news exposure, polarization, and network homophily over the different methods used for their measurement.

1 Deviations from the protocol

The volume of papers our query returned prevented an in-depth analysis of confounding variables. Instead, our assessment of quality relied on the sampling strategy and sampling strategy and sample size, the method used, sources of heterogeneity and transparency criteria, like open data practices and pre-registration. Furthermore, we were able to construct the co-author network by matching the author's names, but unable to produce a meaningful co-citation network due to the incompleteness and ambiguity of references in the export format that we used.

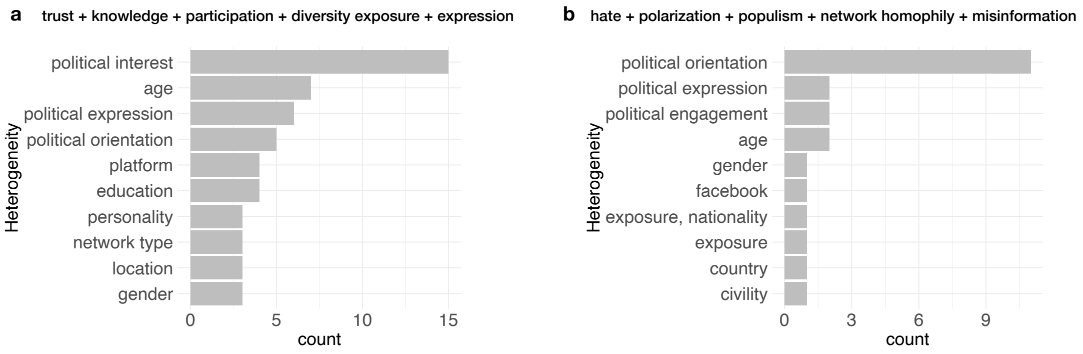

Supplementary Figure 3: Moderator variables reported in studies within the review sample. (a) Reported sources of effect heterogeneity for studies with major outcome measures that are beneficial for democracy (trust, knowledge, participation and diversity of exposure). For example, the effect of digital media on political knowledge (or the relationship between the two variables) was moderated by political interest in 21 studies. (b) Most prominent moderator variables reported in studies with outcome measures that are detrimental for democracy (hate, polarization, populism, network homophily. For example, when the effect of digital media on polarization was moderated by political orientation, the effect varied (in strenth or directon) between people with different political orientation.

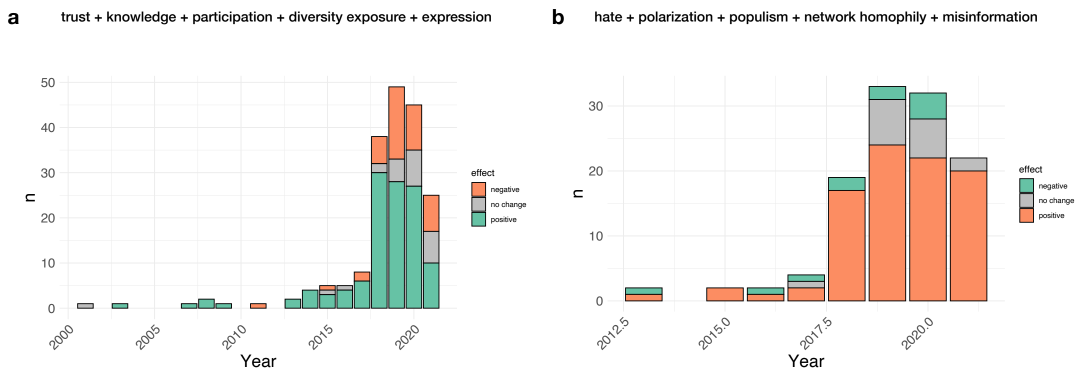

Supplementary Figure 4: Number of studies published over time by effect direction. Colour representing effect valence with regard to democracy (green as beneficial, red as detrimental for democracy). (a) effects of studies published with outcome measures that are beneficial for democracy (trust, knowledge, participation and diversity of exposure). (b) effects of studies published with outcome measures that are detrimental for democracy (hate, polarization, populism, network homophily, misinformation). For both categories of outcome variables, authors found mostly statically positive relationships, that means, amplifications of positive but also negative phenomena through digital media.

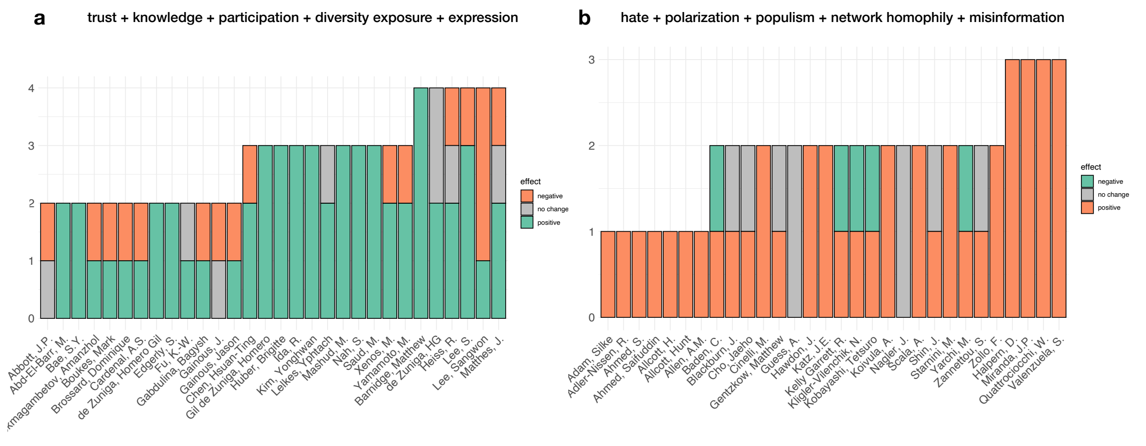

Supplementary Figure 5: Number of studies published by authors, by effect direction. Colour representing effect valence with regard to democracy (green as beneficial, red as detrimental for democracy). (a) effects of studies published with outcome measures that are beneficial for democracy (trust, knowledge, participation and diversity of exposure). (b) effects of studies published with outcome measures that are detrimental for democracy (hate, polarization, populism, network homophily, misinformation). For both categories of outcome variables, authors found mostly statically positive relationships, that means, amplifications of positive but also negative phenomena through digital media. We do not find strong patterns for field dominating authors.

Supplementary Table 1: Study selection criteria

Exclusion criteria	Inclusion criteria
Evidence synthesis (meta analyses, reviews)	Digital media variable
Conceptual and theoretical work	Political outcome variable
Other non original empirical work	Original empirical work
Simulation studies	
Studies published in other languages than English	
Abstracts or extended abstracts	
Preprints and unpublished work	
Conference submissions	
Any papers clearly not matching the subject focus (e.g. papers on polarisation phenomena in physics)	
Purely methodological papers (e.g. hate speech or misinformation detection approaches)	
Studies on the digitization of government and administration	

Note. All inclusion criteria have to be met ("and"-connector for inclusion criteria), if one of the exclusion criteria applies, the study is excluded from the review sample ("or"-connector for exclusion criteria).

Supplementary Table 2: Inter Rater Reliability

Variable	Percentage of agreement	Krippendorff's Alpha
Digital media	91.18	0.84
Political outcome	79.41	0.75
Effect	85.29	0.75

Note. Digital media categories: social media, internet, other; top 10 political outcome variables: participation, polarization, trust, knowledge, expression, exposure, misinformation, echo chamber, populism, hate; effect for democracy: beneficial, detrimental, no effect.

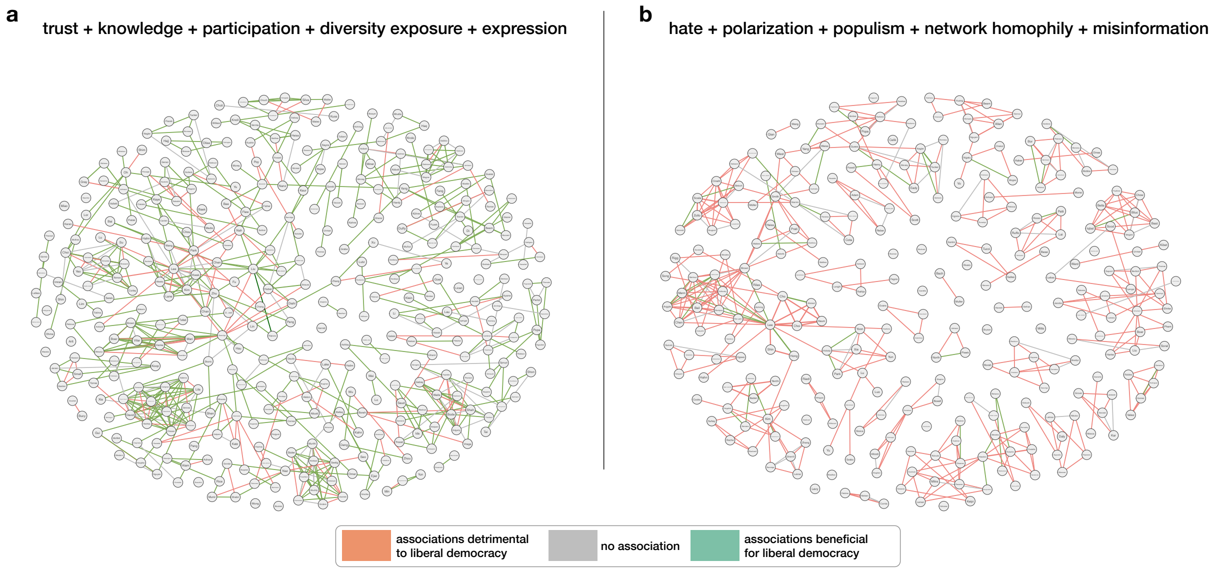

Supplementary Figure 6: Co-author network from the sample, a link between two authors represents a co-authored paper in our sample. Visualization is using a spring-layout, showing authors spatially closer together when they are connected.

Causality vs. Correlation: A Brief Primer

The 'fundamental problem of causal inference' is the impossibility to observe the effects of a variable on a specific individual. To measure *individual* causal treatment effects, one would have to measure both, the actual state of an individual under treatment (the reality) but at the same time, the counterfactual — the state of the same individual had they not been treated [1]. Perfect experiments permit the observation of *average* causal treatment effects by comparing the outcomes of treatment and control groups, with the groups made equal on all variables other than the treatment through random assignment. Usually, in the absence of randomized treatment assignment with observational data, such as survey data, the identification of causal effects is impossible due to the fact that individuals differ systematically on variables other than the treatment (or independent) variable. For example, selection effects are among the most common sources of non-causal explanations of correlations. Selection bias means that as the treatment and control groups differ systematically because only specific individuals (e.g. those with a specific media preference, say, watching FOX) select into the (not randomly selected) treatment group. Therefore, one cannot conclude much about the causal effects of watching FOX as people who do may differ on many other dimensions from people who prefer to watch, say, CNN. Issues of reverse causality (the outcome causing the independent variable and not vice versa) and heterogeneous treatment effect bias (the independent variable having differing effects for different groups of individuals) are other common threats to causal inference. Therefore, the interpretation of observational evidence needs to be more cautious as the observed associations can be bi-directional; they can be confounded by third variables or the associations can have other, unobserved causes. Yet, under certain conditions, it is possible to rule out non-causal explanations for associations, even in studies without random assignment that report observational data (see the work of this year's laureates of the Nobel Memorial Prize in Economics [2–4]. We summarize the fundamental logic of the dominant causal inference methods used in papers reported in this review in Fig. 2 of the main paper.

References

1. Rubin, D. B. Causal inference using potential outcomes: Design, modeling, decisions. *Journal of the American Statistical Association* **100.** Publisher: Taylor & Francis, 322–331 (2005).
2. Of Sciences, T. R. S. A. *The Sveriges Riksbank Prize in Economic Sciences in Memory of Alfred Nobel 2021* en-US. 2021. (2021).
3. Angrist, J. D. & Pischke, J.-S. *Mostly harmless econometrics* (Princeton university press, 2008).
4. Angrist, J. D. & Pischke, J.-S. The credibility revolution in empirical economics: How better research design is taking the con out of econometrics. *Journal of economic perspectives* **24,** 3–30 (2010).
